# Supplementary material for: mRNA Capture Sequencing and RT-qPCR for the Detection of Pathognomonic, Novel, and Secondary Fusion Transcripts in FFPE Tissue: A Sarcoma Showcase
Source: Int J Mol Sci. 2022 Sep 20;23(19):11007. doi: 10.3390/ijms231911007 (PMC9569610; doi:10.3390/ijms231911007)

Supplemental figure S2.

cohort II - P17 - 100x

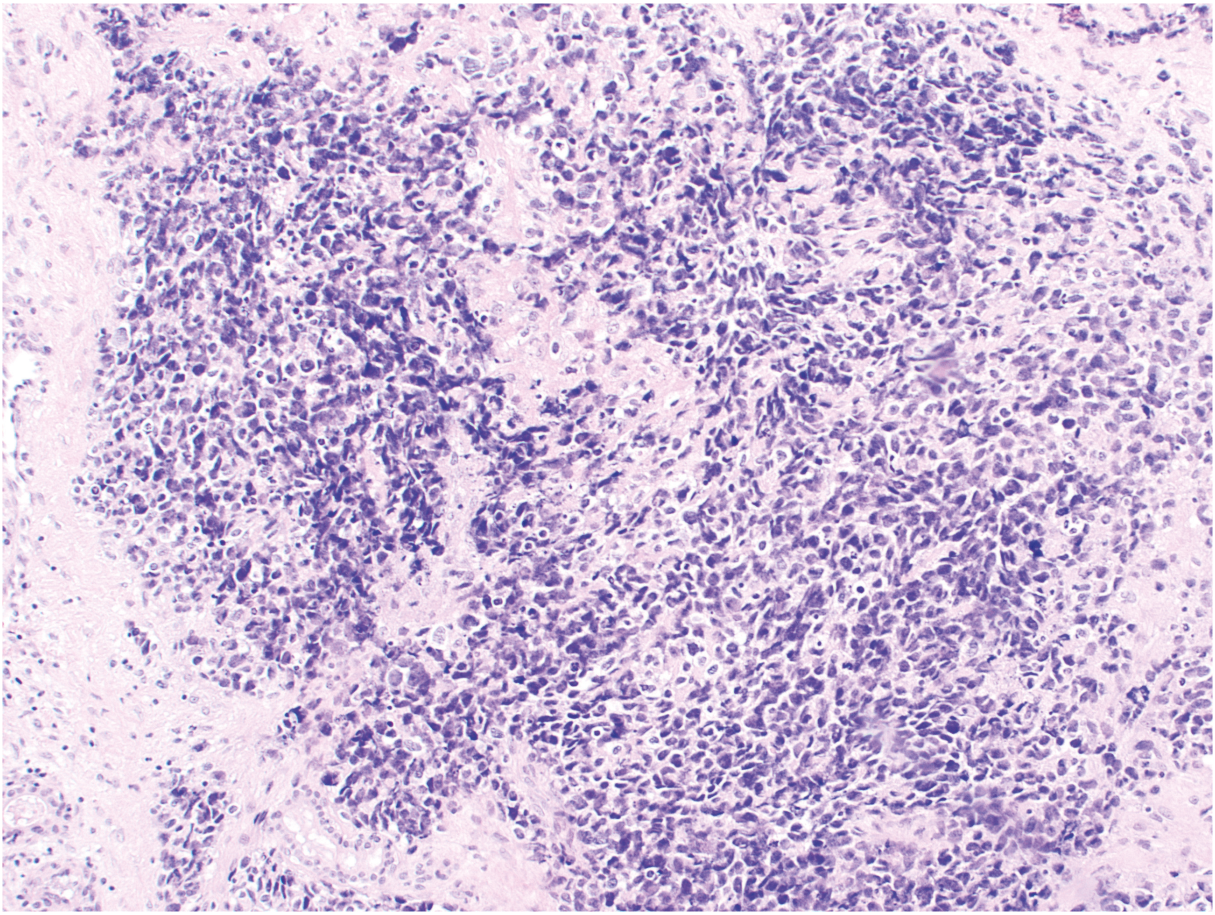

cohort II - P18 - 100x

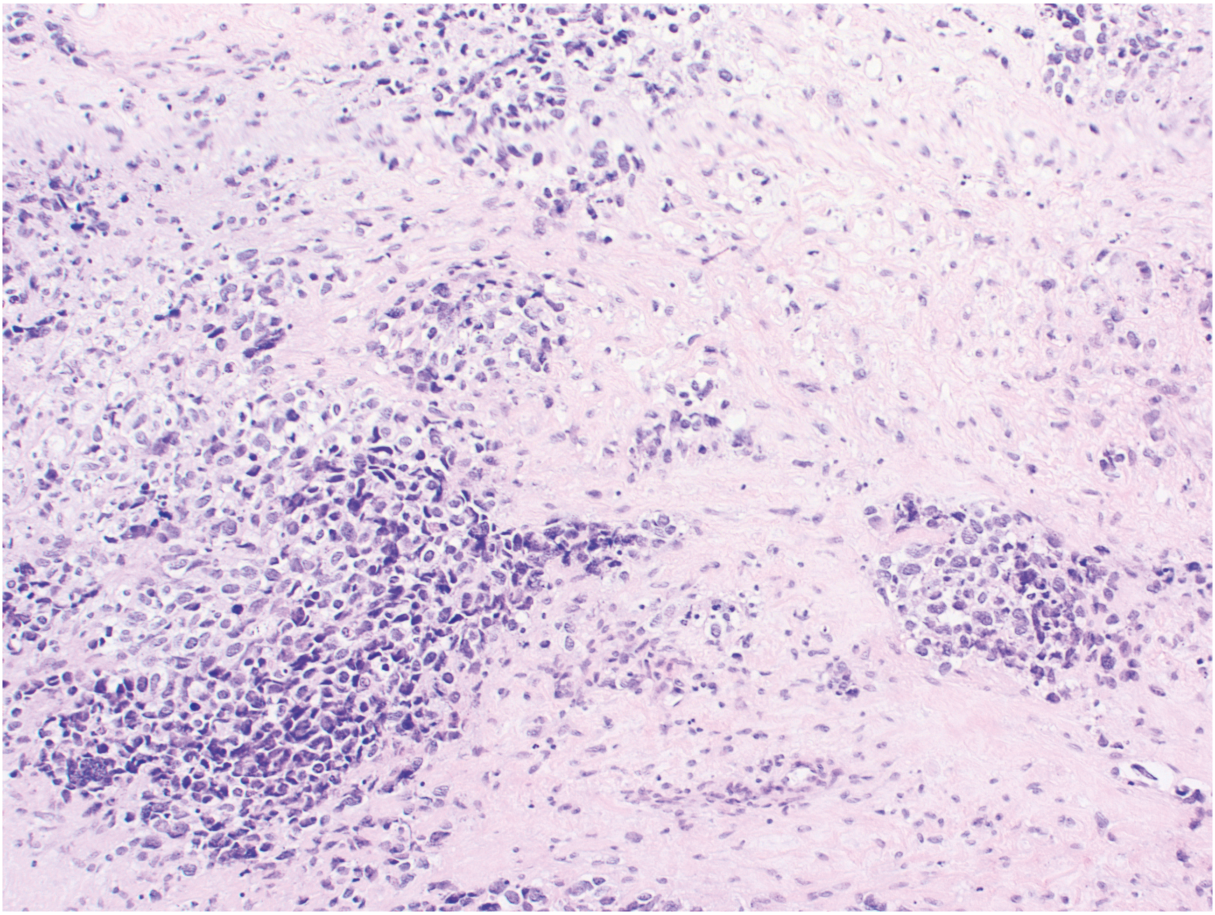

cohort II - P19 - 100x

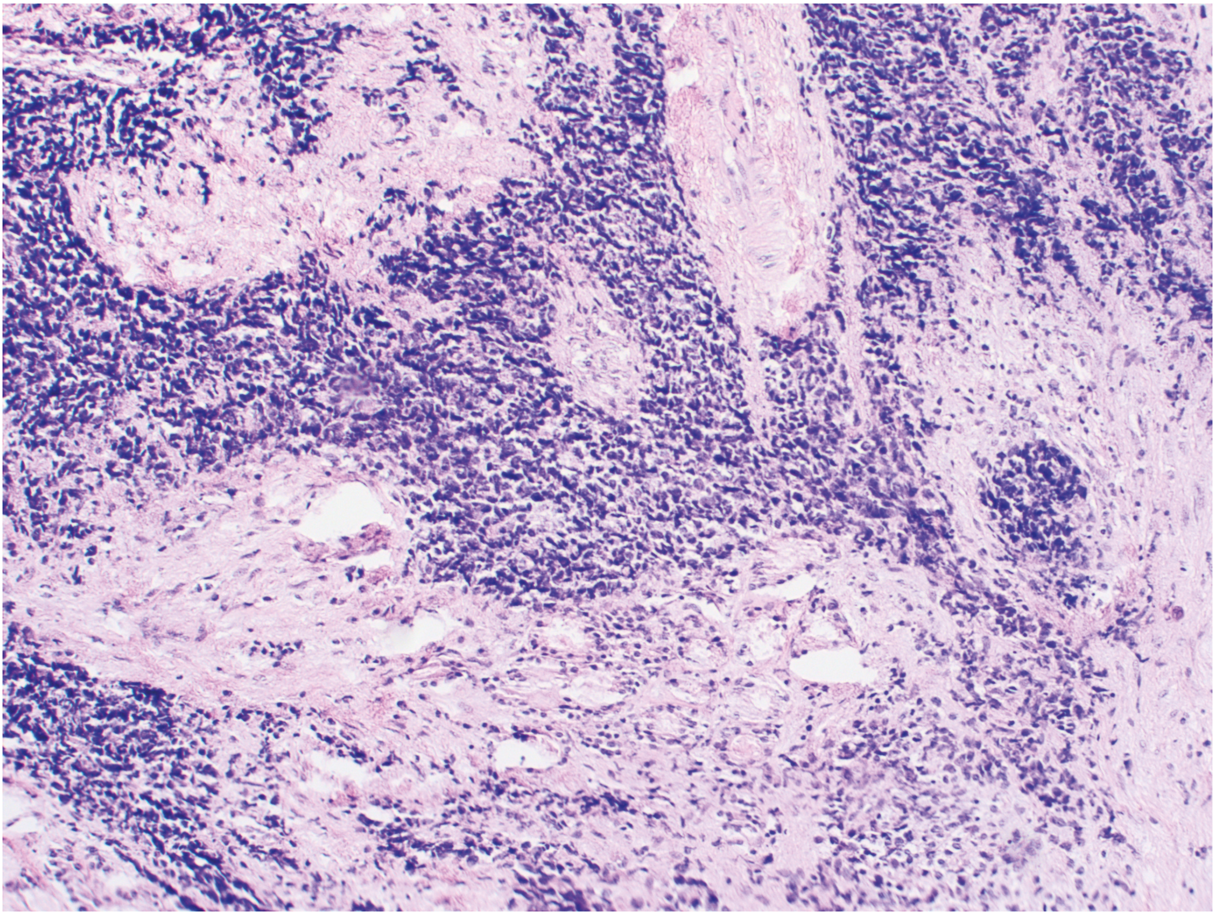

cohort II - P20 - 100x

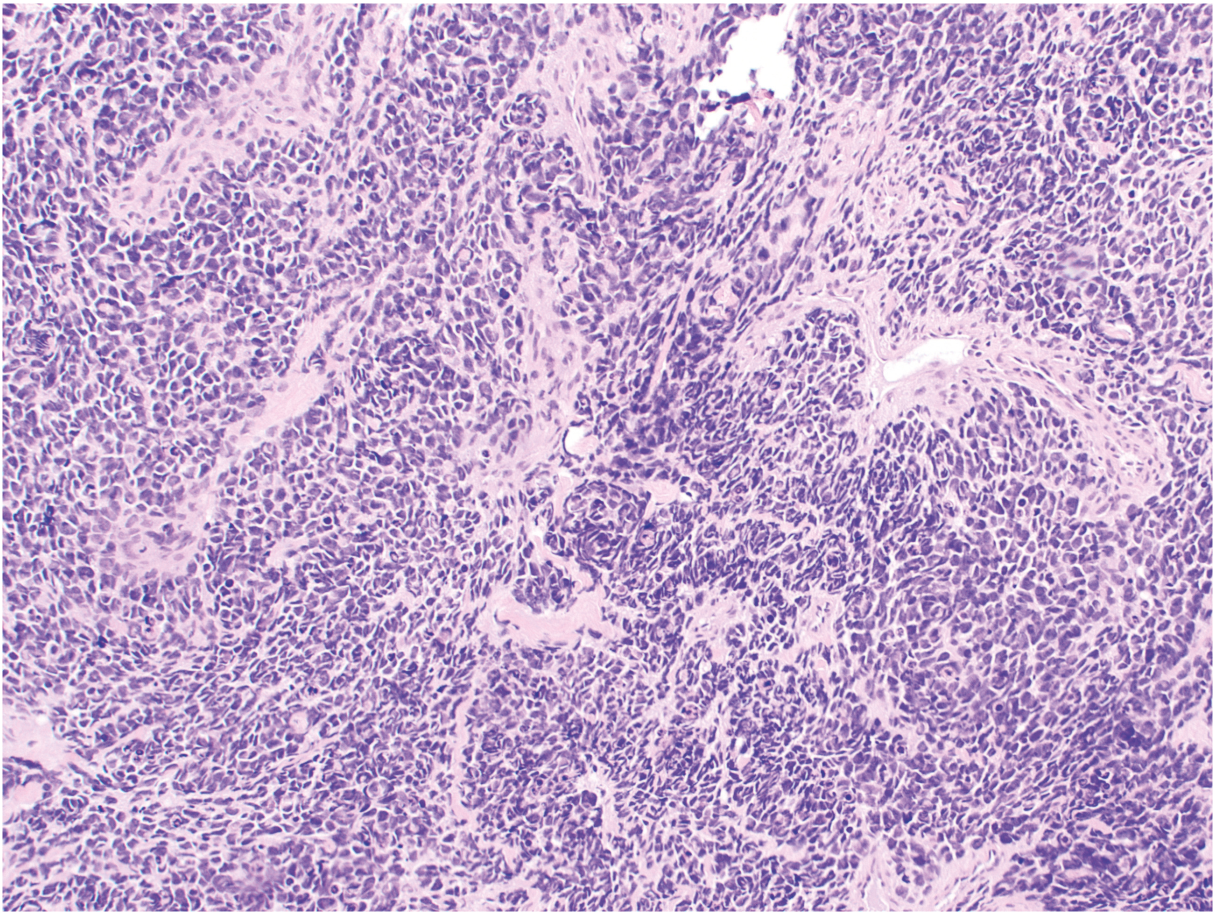

cohort II - P21 - 200x

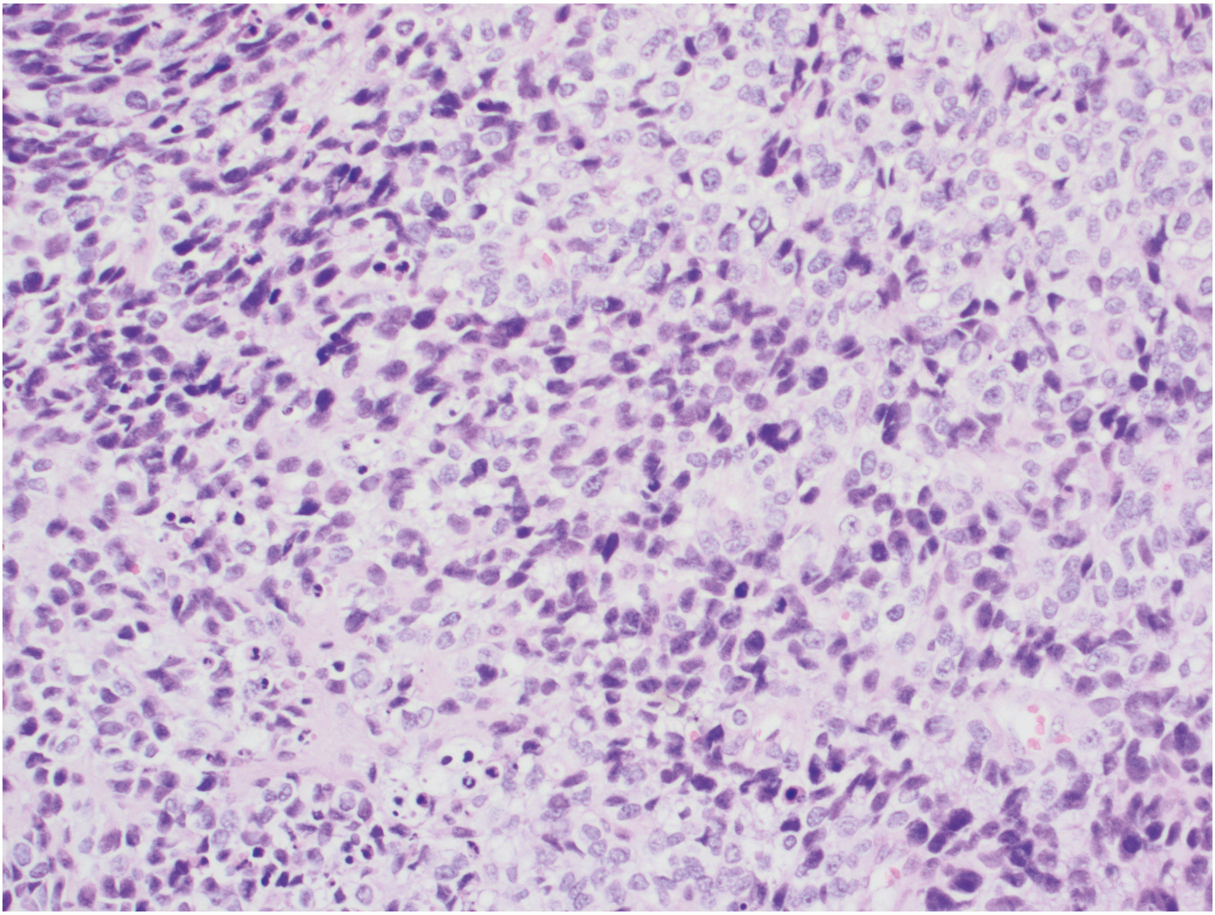

cohort II - P22 - 100x

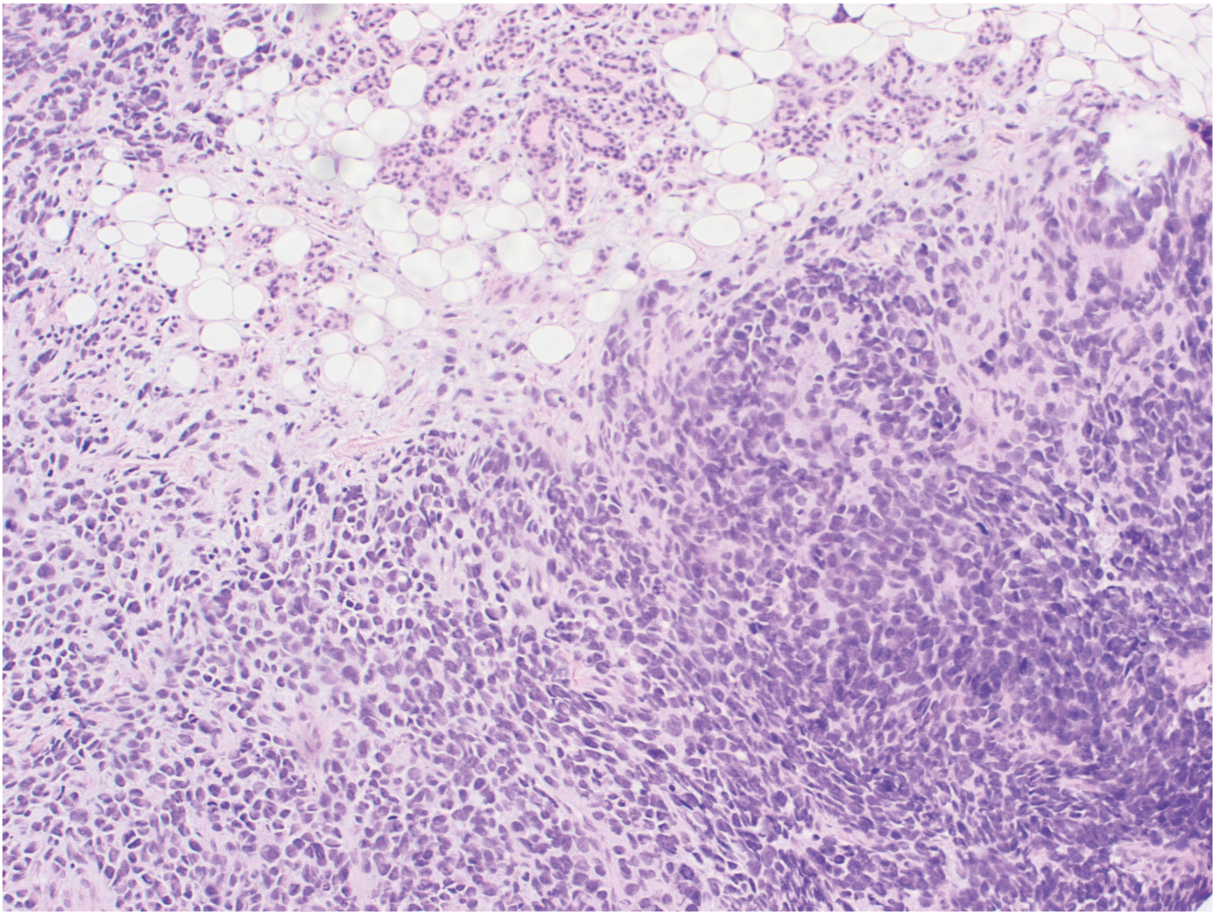

cohort II - P23 - 100x

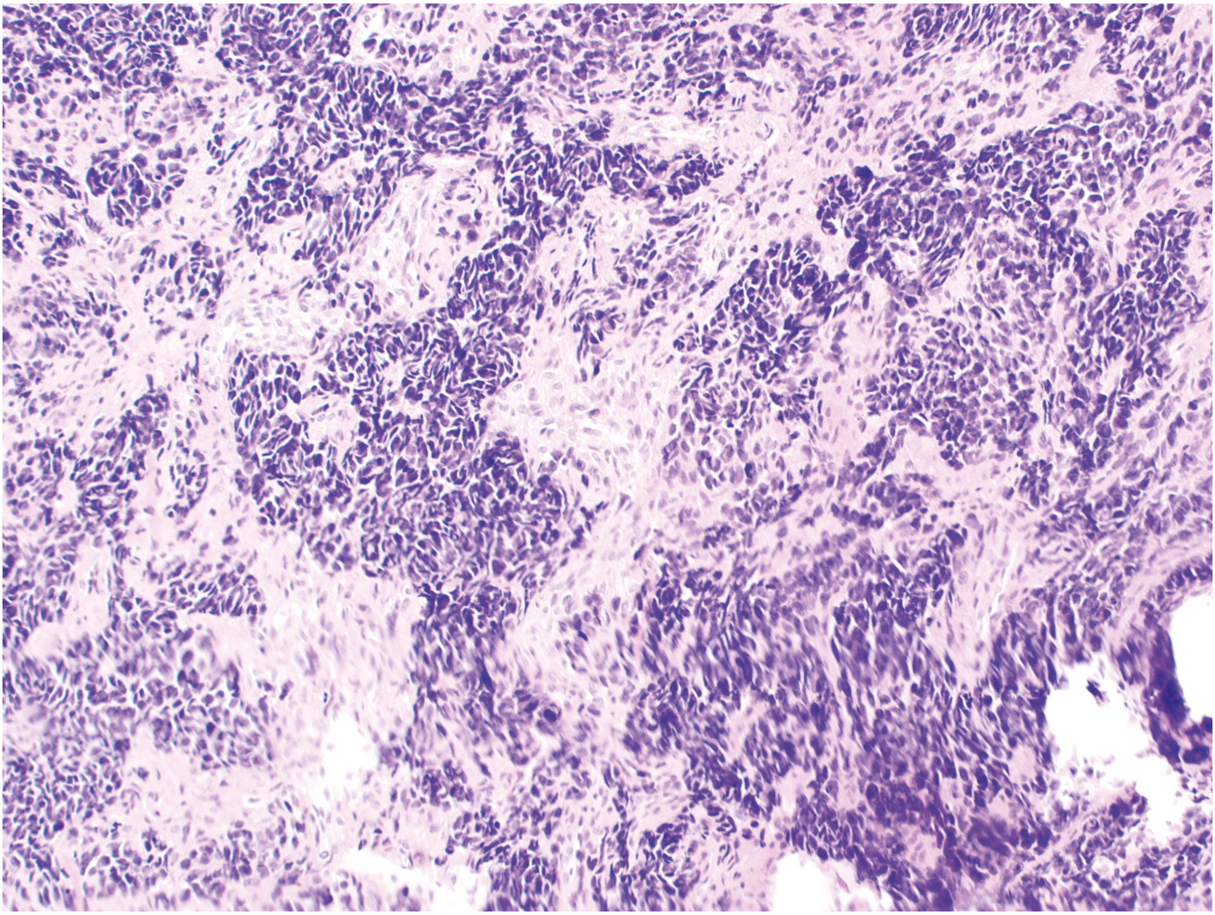

cohort II - P24 - 200x

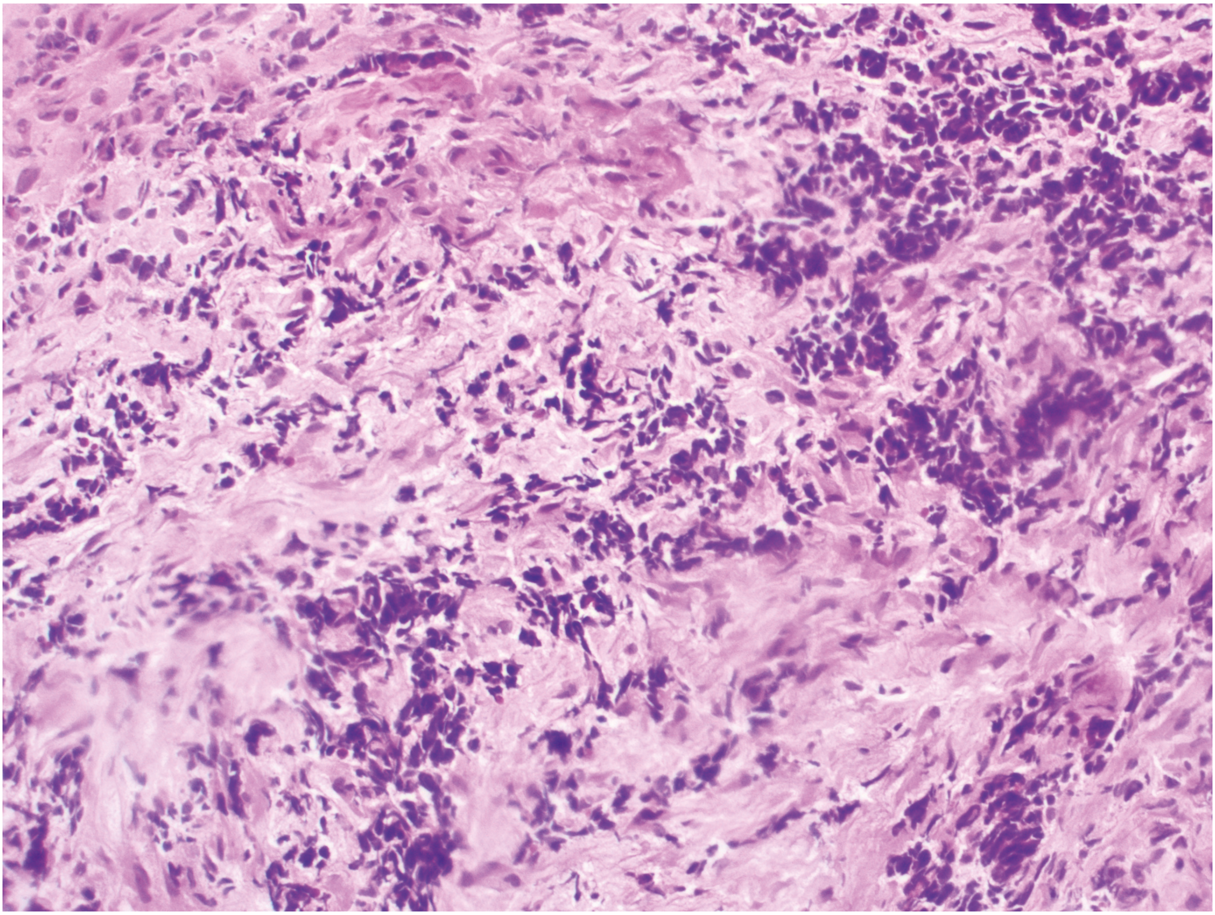

cohort II - P25 - 100x

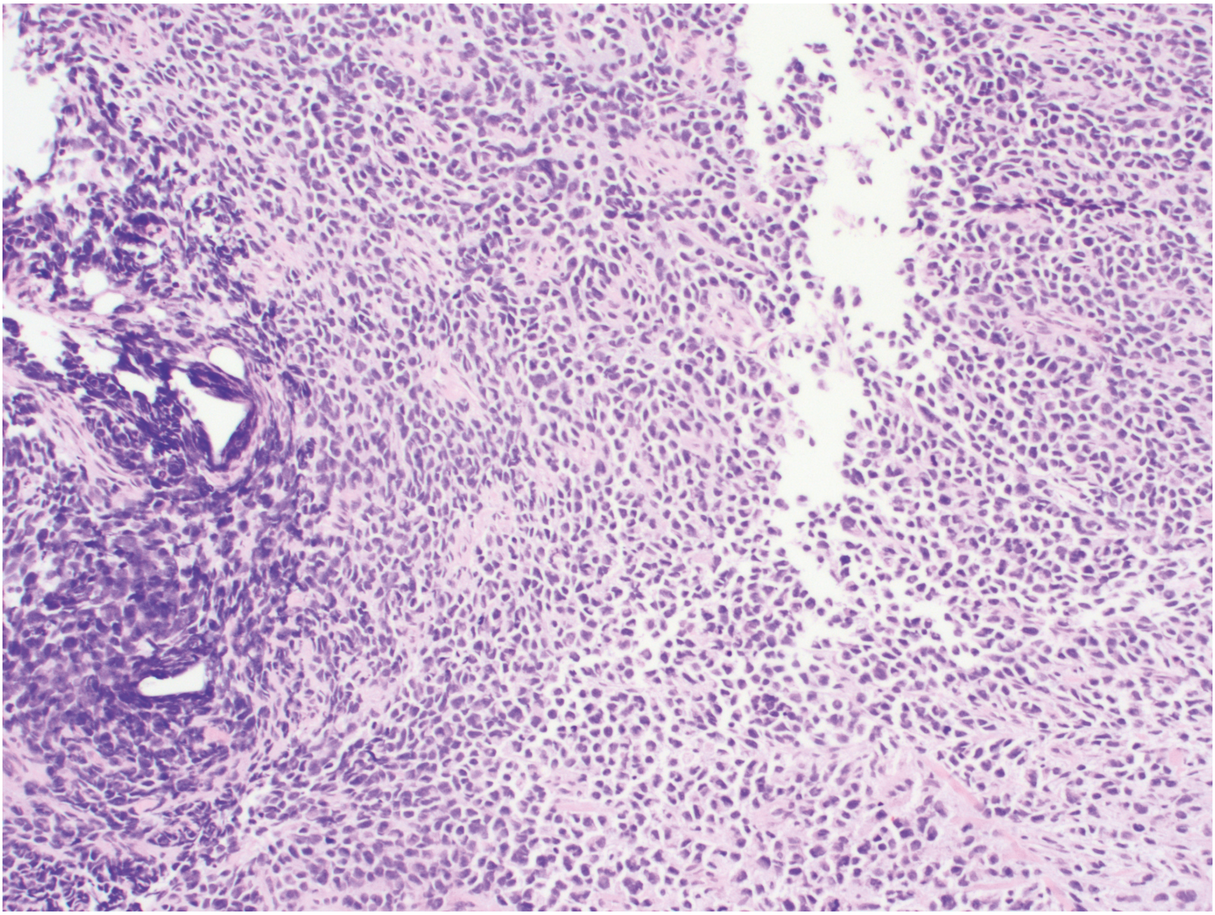

cohort II - P26 - 200x

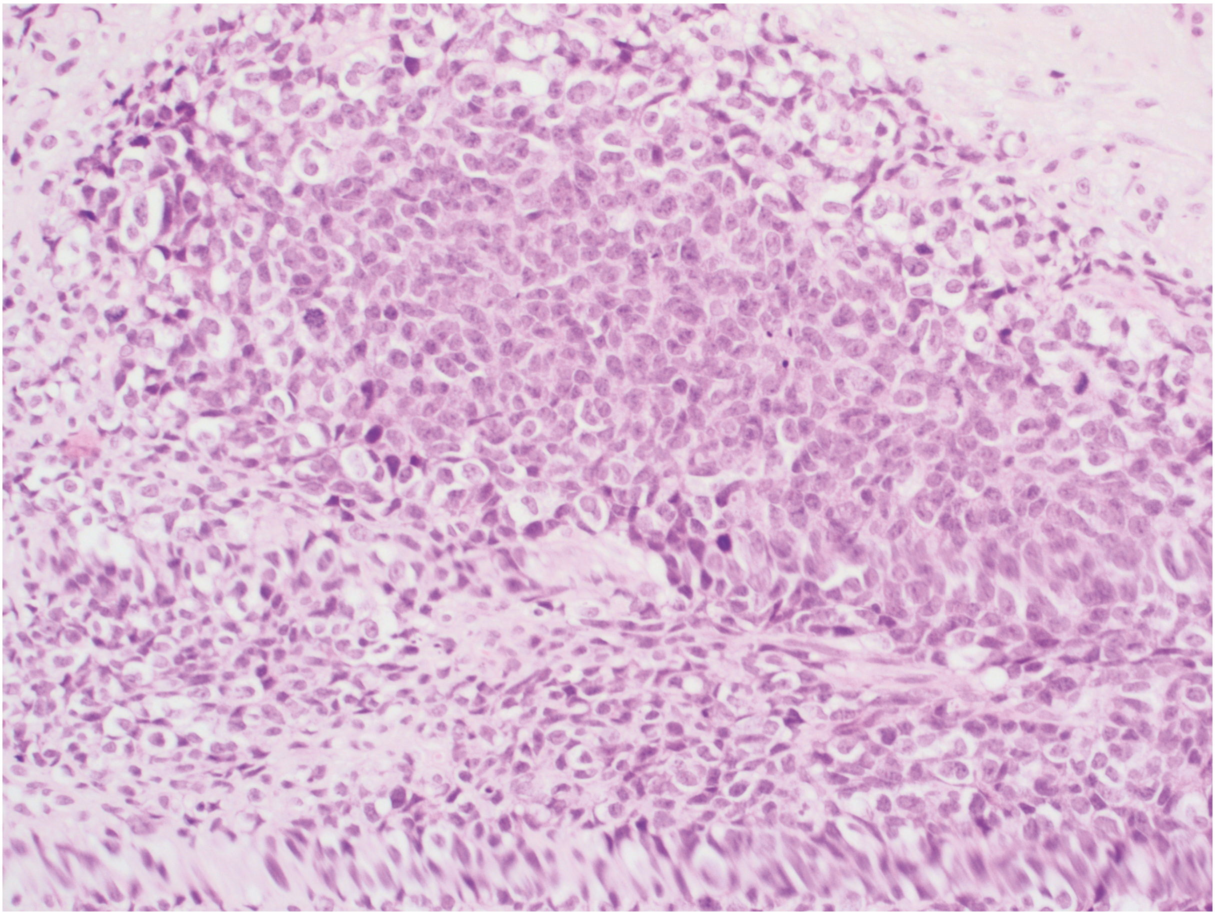

cohort II - P27 - 100x

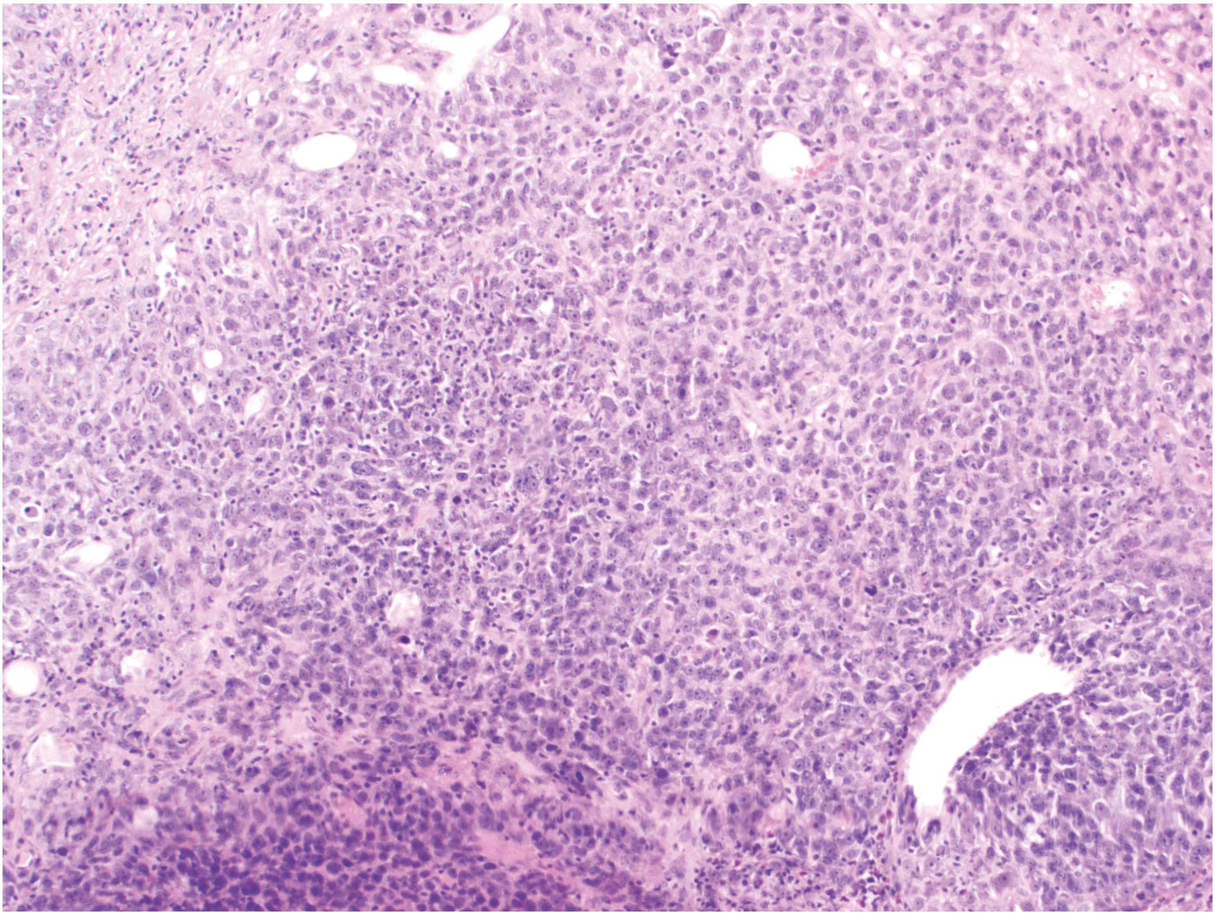

cohort II - P28 - 100x

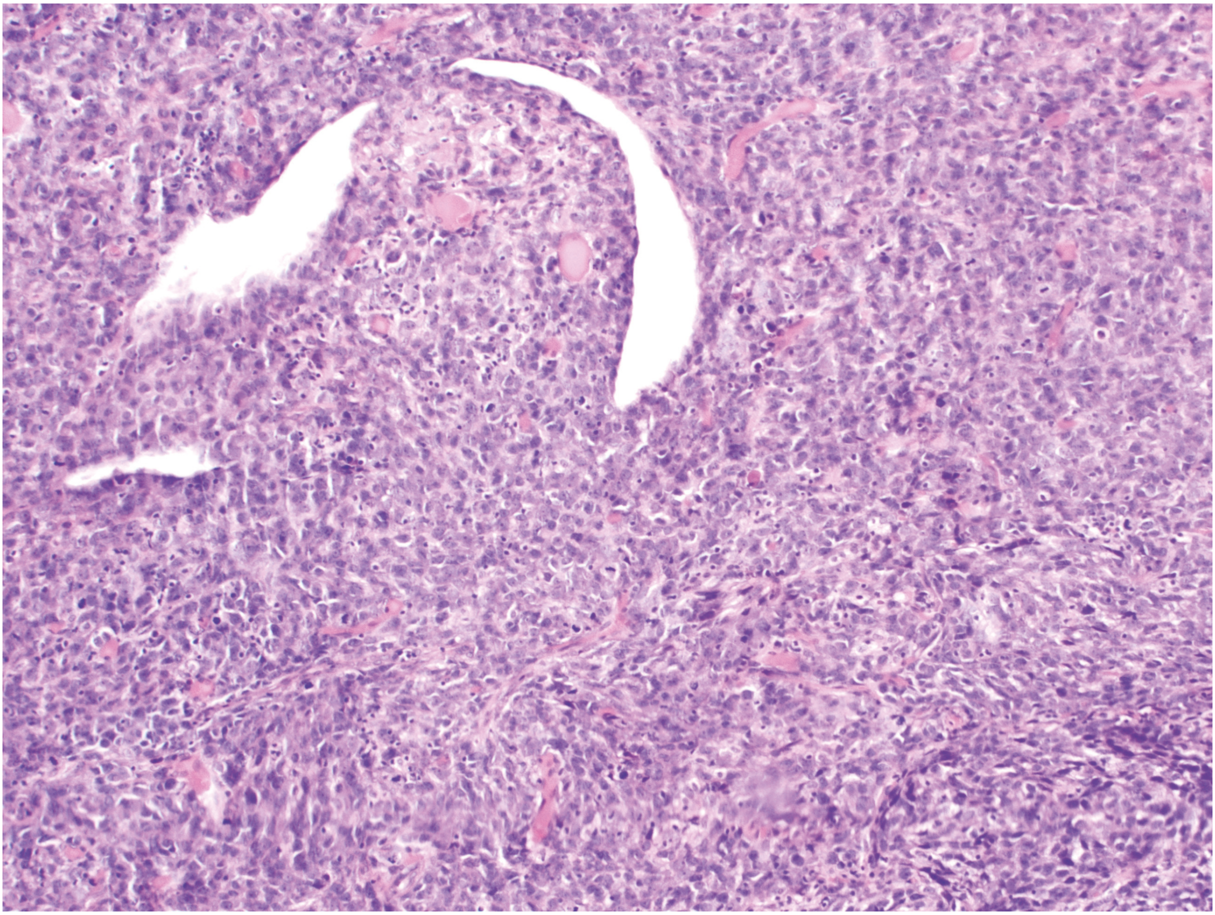

cohort II - P29 - 200x

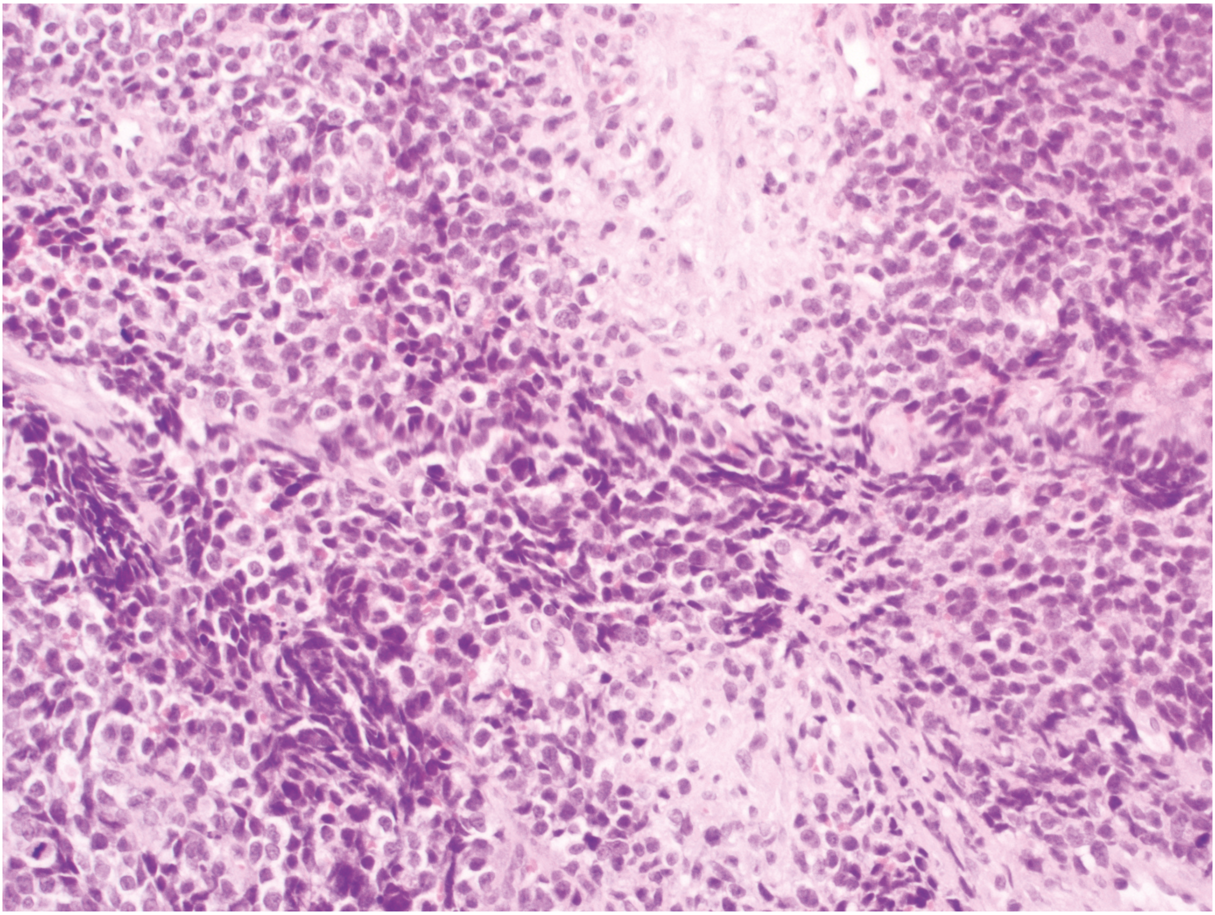

cohort II - P30 - 200x

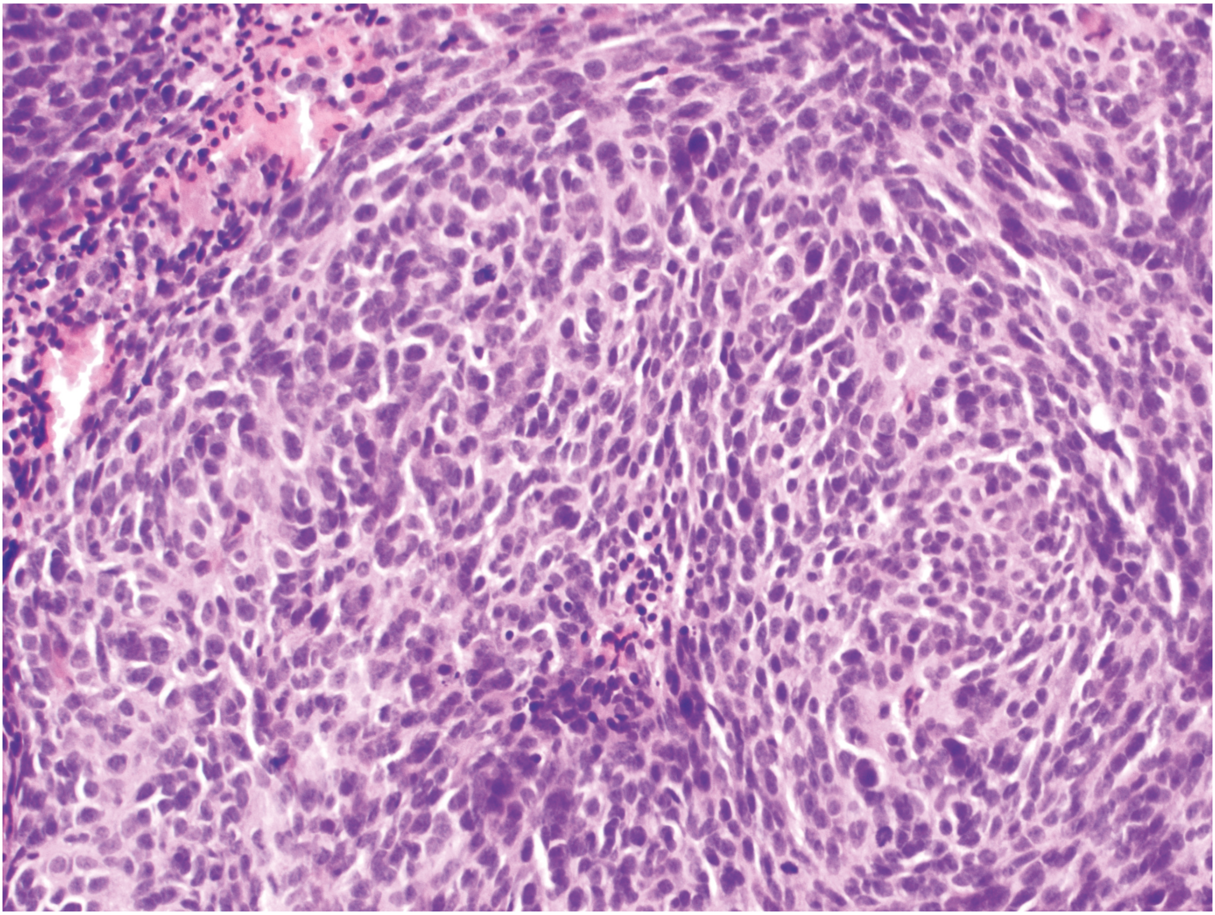

cohort II - P31 - 200x

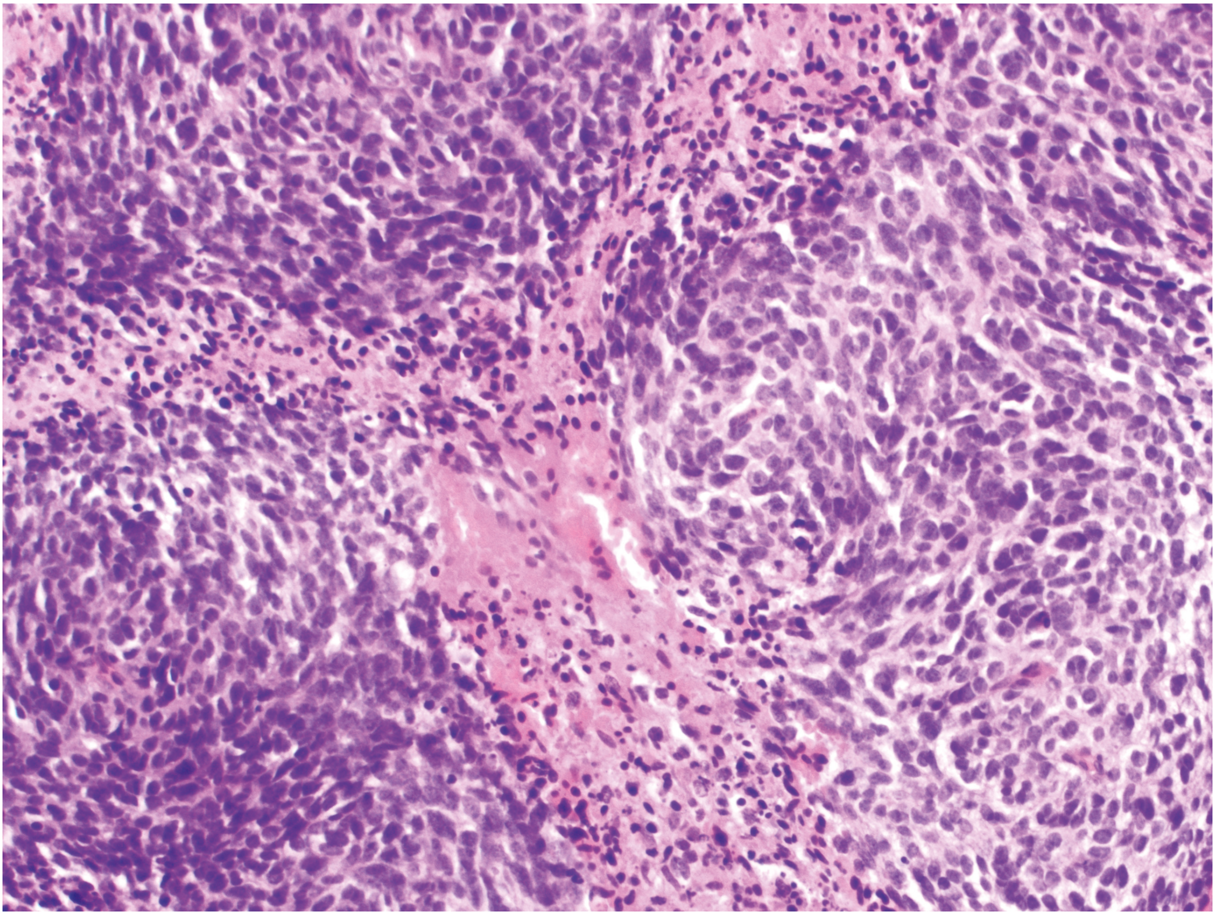

cohort II - P32 - 200x

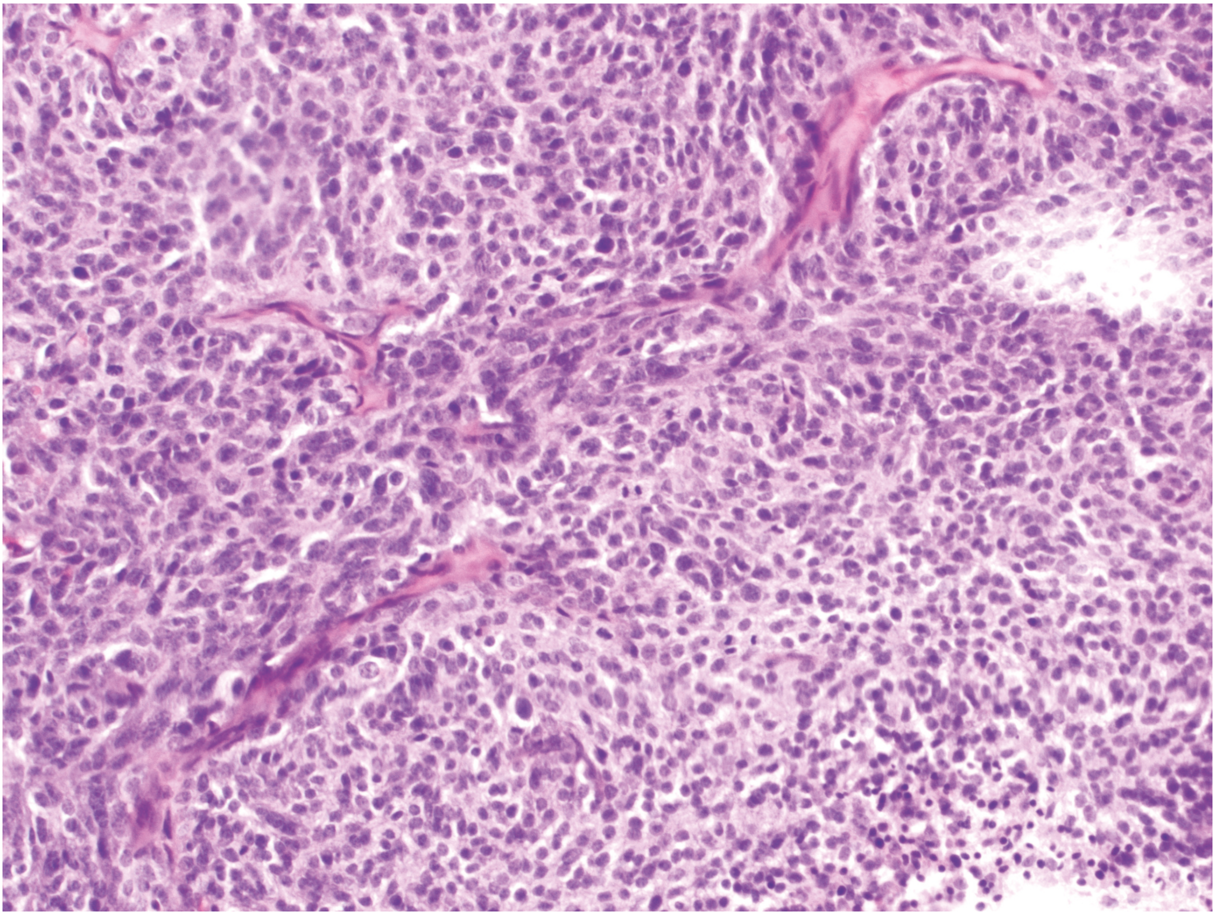

cohort II - P33 - 200x

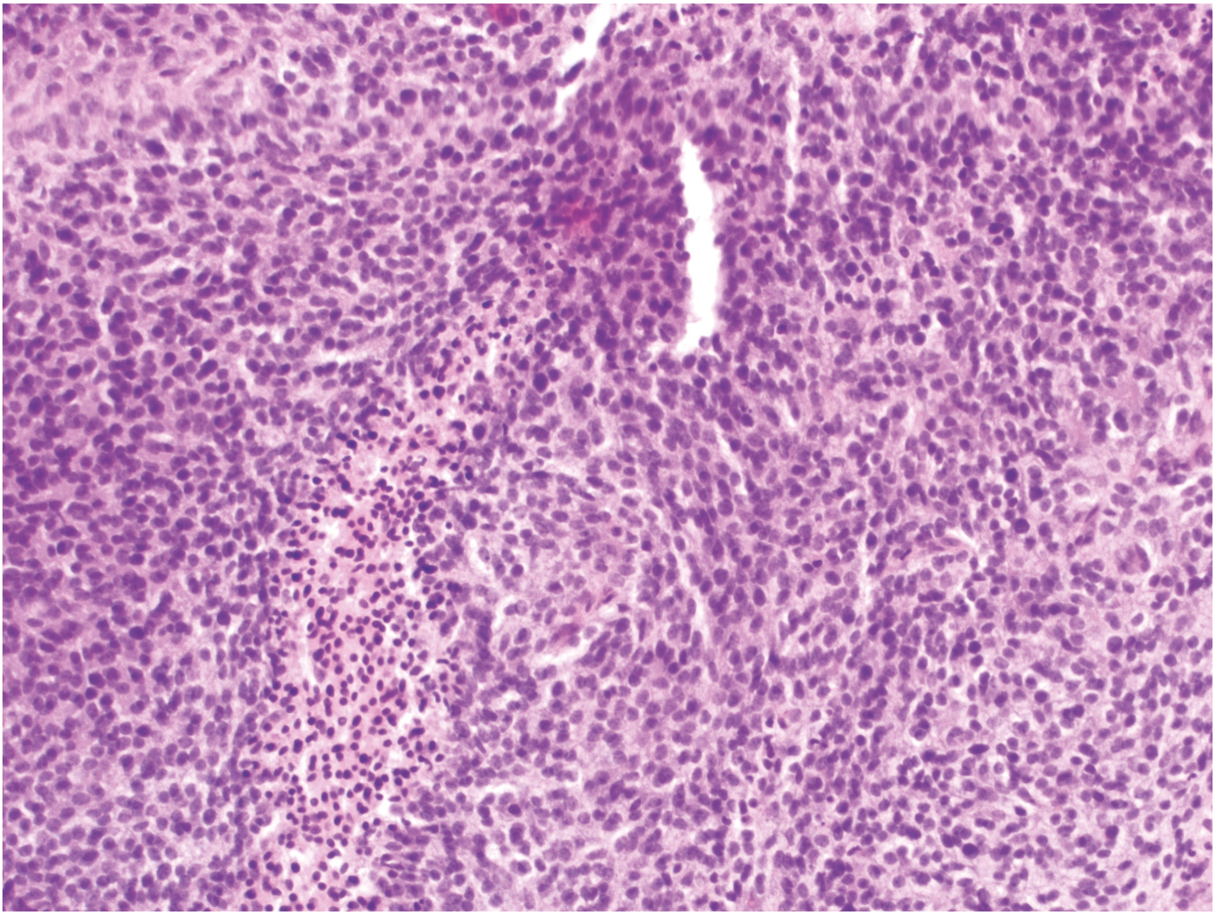

Supplement: Supplementary file 1 [file ijms-23-11007-s001.zip › Figure S2.pdf]
