# Supplementary figures and images for: mRNA Capture Sequencing and RT-qPCR for the Detection of Pathognomonic, Novel, and Secondary Fusion Transcripts in FFPE Tissue: A Sarcoma Showcase
Source: Int J Mol Sci. 2022 Sep 20;23(19):11007. doi: 10.3390/ijms231911007 (PMC9569610; doi:10.3390/ijms231911007)

# Supplemental figure S1.

cohort I - P11 - 100x

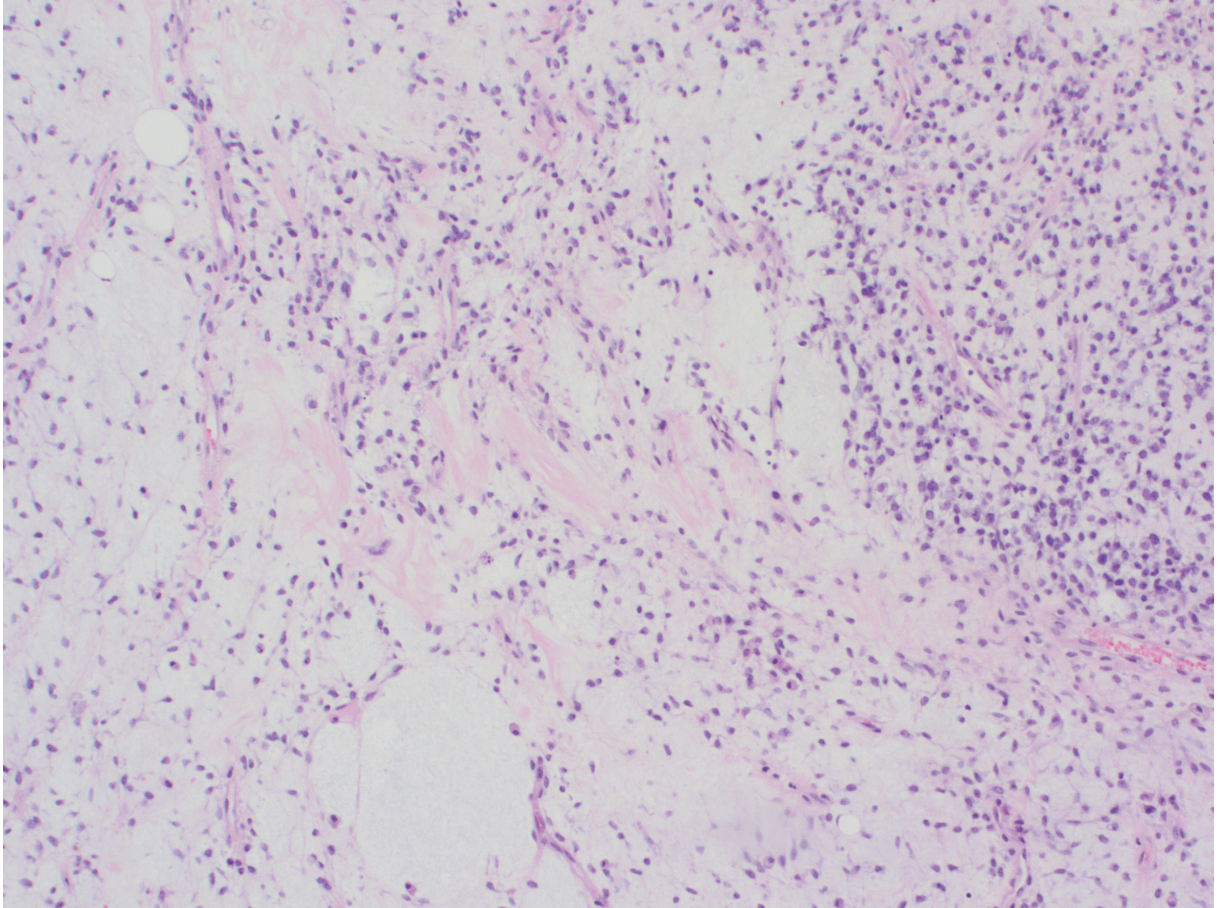

cohort I - P12 - 200x

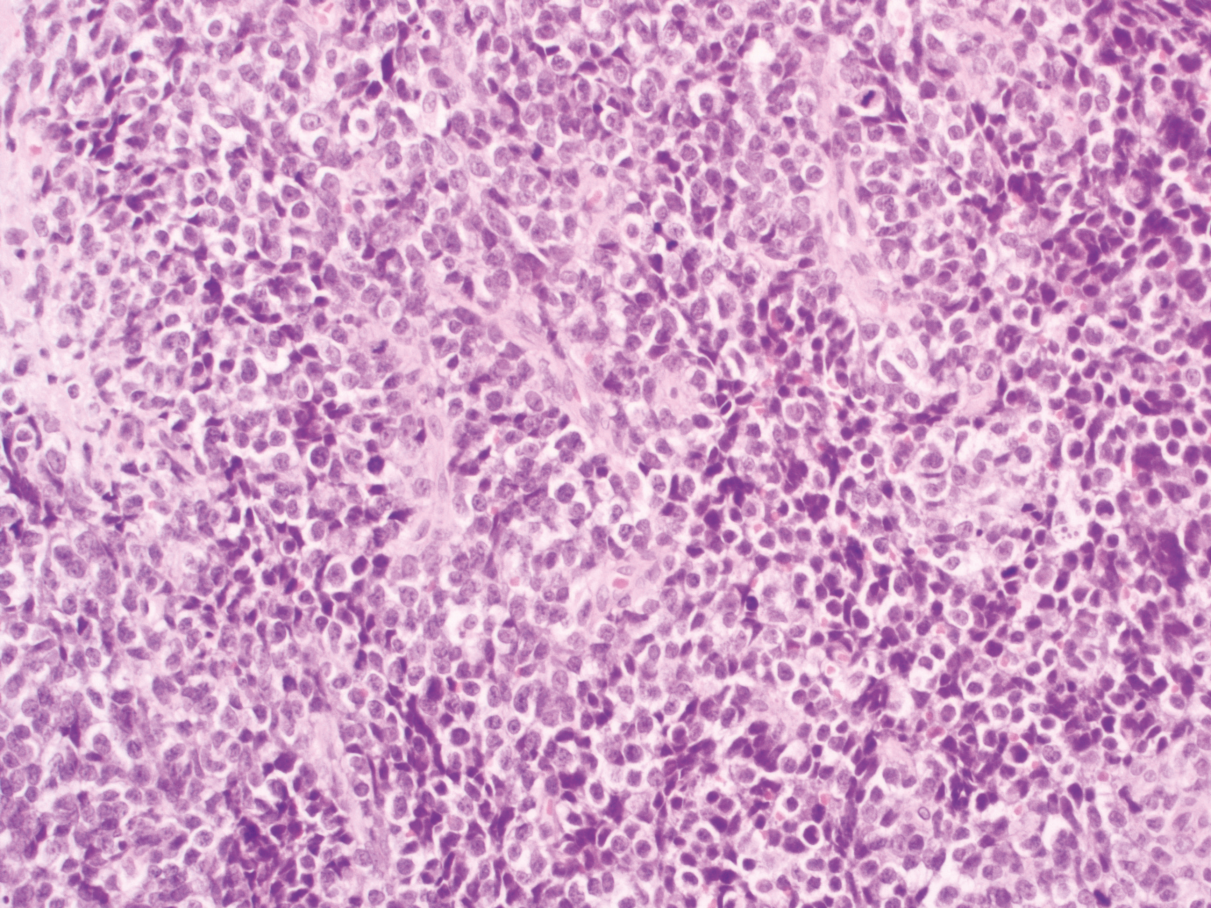

cohort I - P13 - 100x

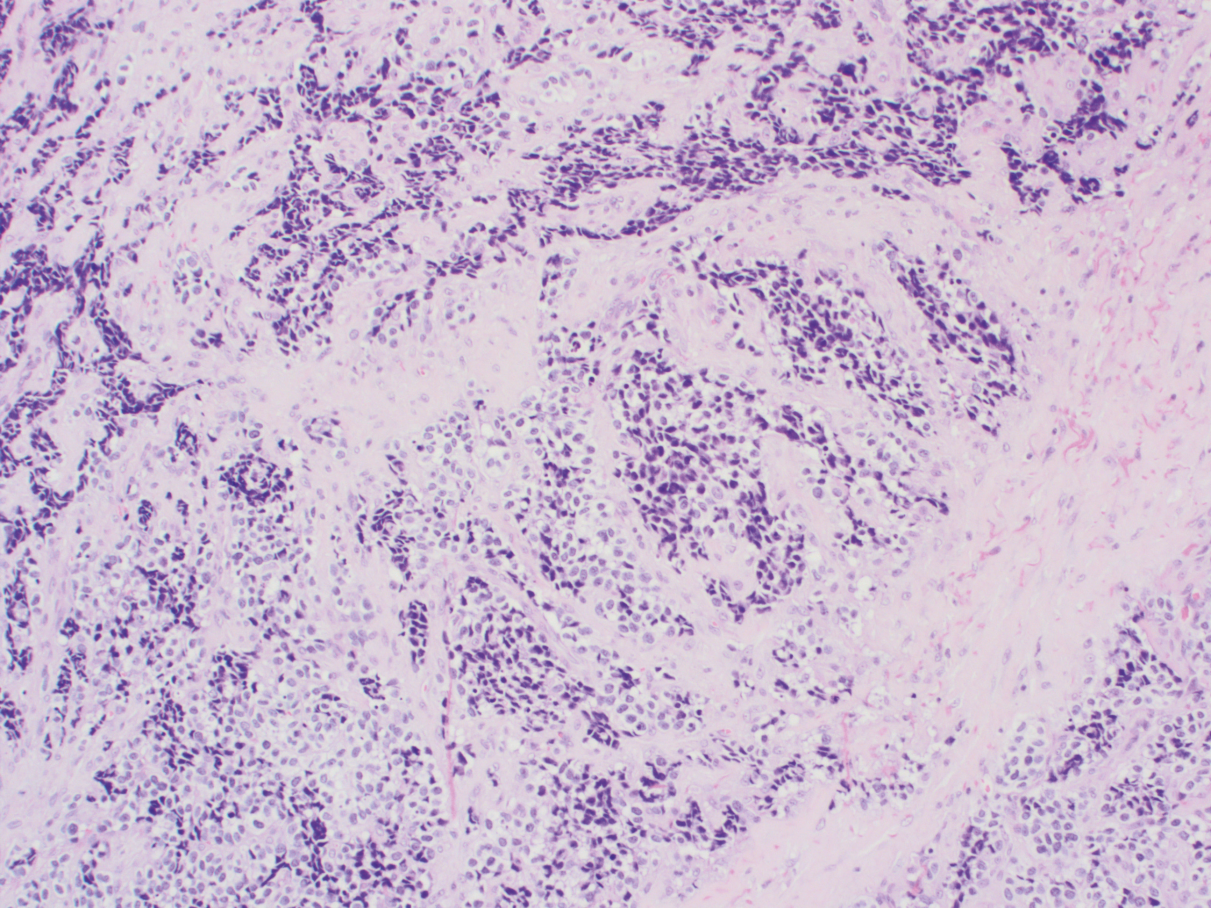

cohort I - P14 - 100x

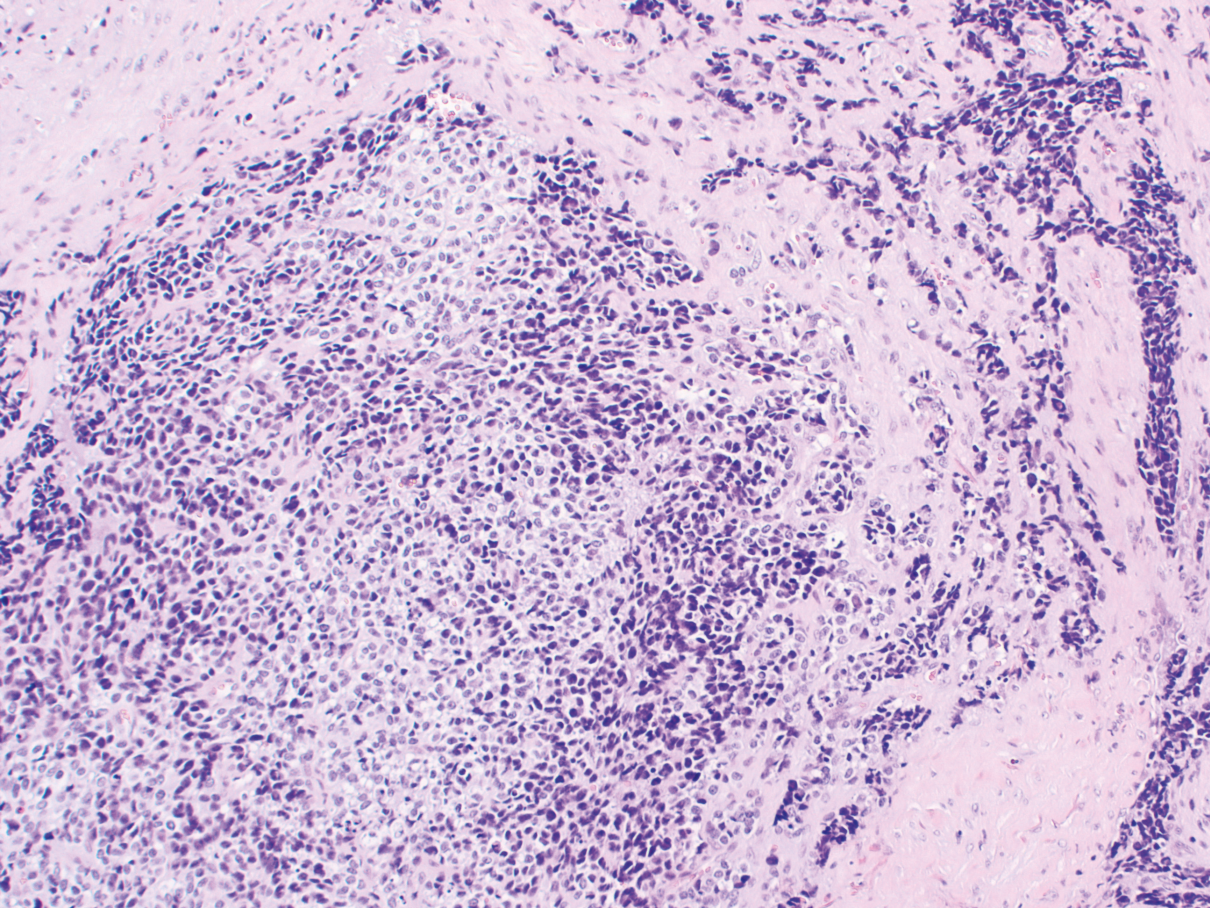

cohort I - P15 - 100x

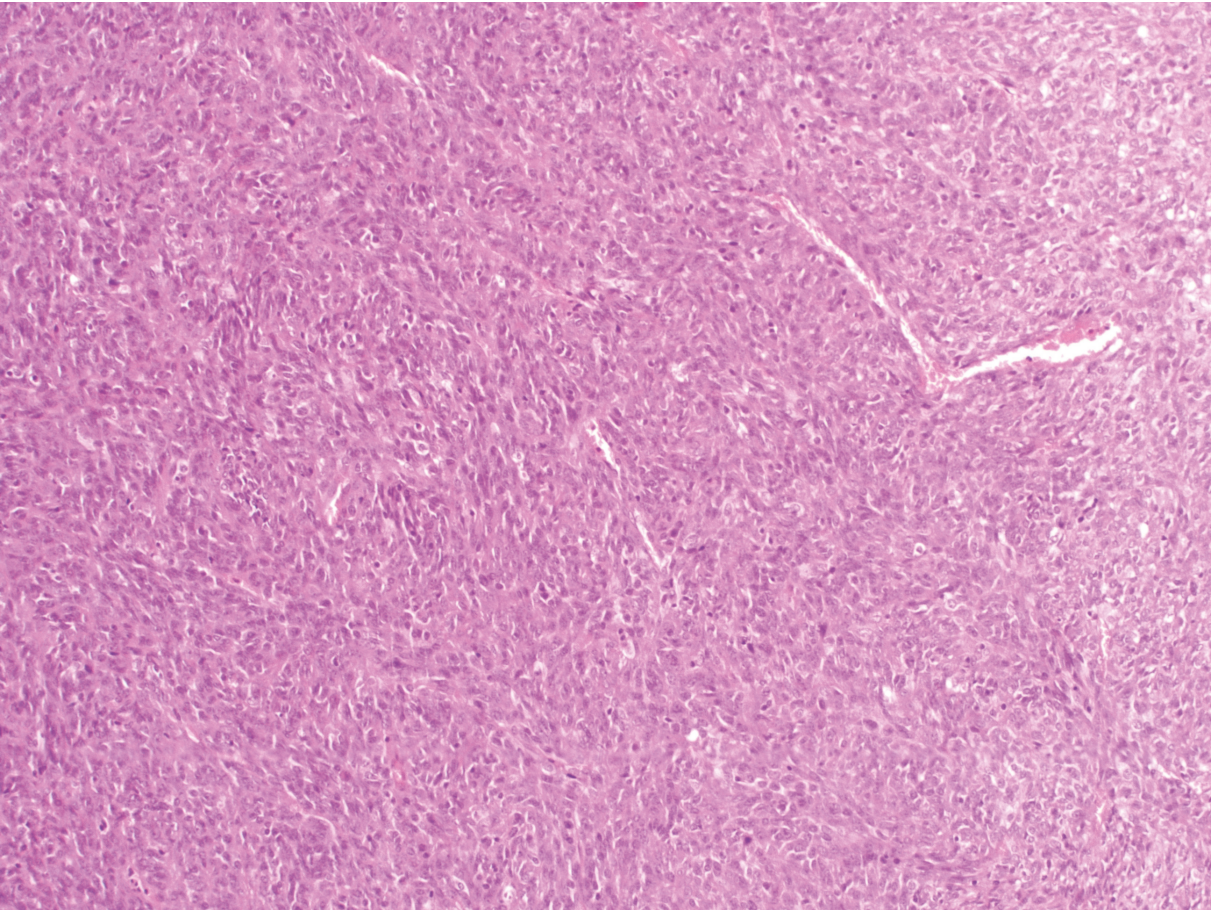

cohort I - P16 - 100x

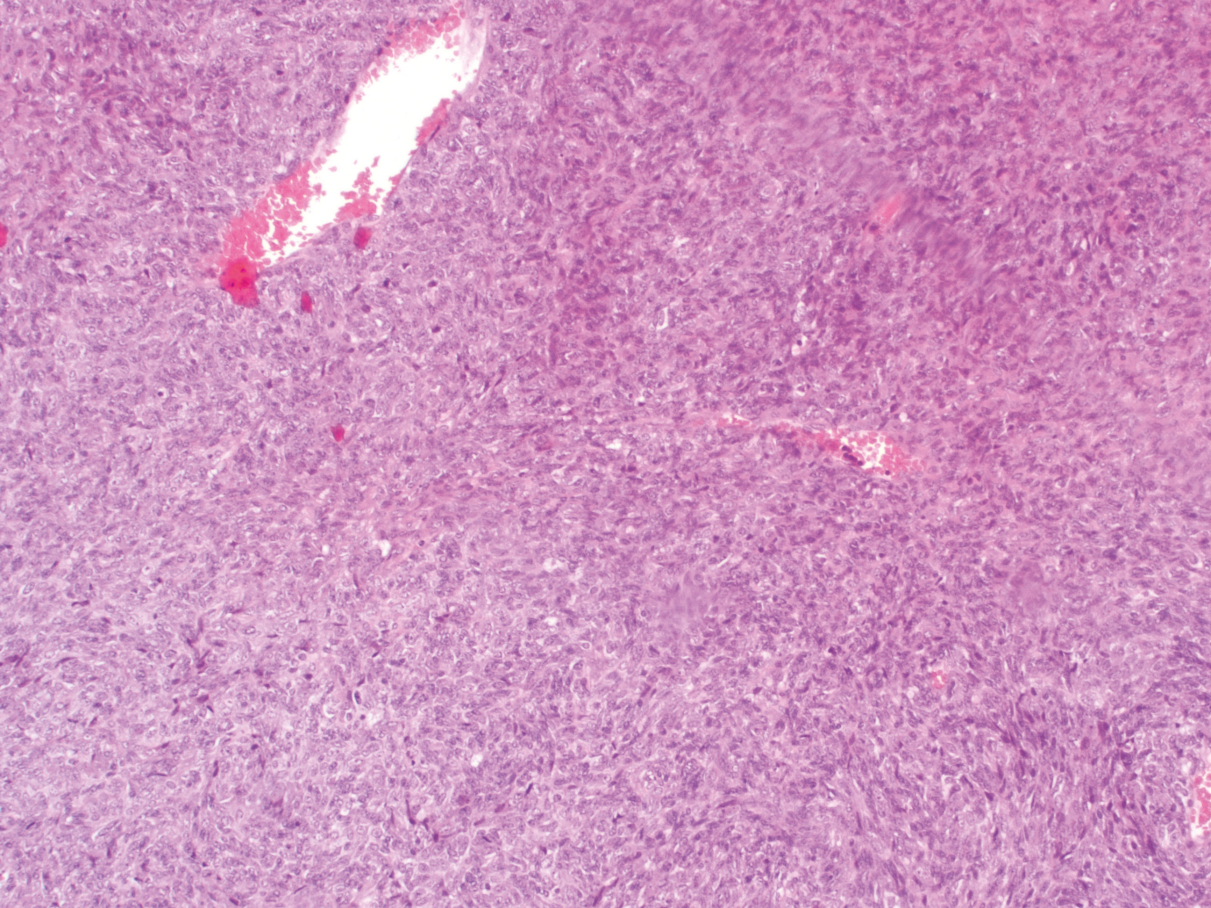

Supplement: Supplementary file 1 [file ijms-23-11007-s001.zip › Figure S1.pdf]
